# Comparative Efficacy and Safety Profile of the Combination of Pulmonary Surfactant and Budesonide vs. Surfactant Alone in the Management of Neonatal Respiratory Distress Syndrome: An Updated Meta-Analysis

**DOI:** 10.3390/medicina61081329

**Published:** 2025-07-23

**Authors:** Urooj Fatima, Naveera Naveed, Zahra Riaz, Emaan Khalid, Aemon Qamer, Shehmeen Baig, Roshaan Fatima, Asawir Hussain, Zoya Mustunsar, Ayesha Khan, Sadia Mangan, Mehak Kumari, Soban Ali Qasim, Ali Hasan, Raheel Ahmed

**Affiliations:** 1 Department of Medicine, Services Institute of Medical Science (SIMS), Lahore 54000, Pakistan; uroojsaif26@gmail.com (U.F.); emaankhalid550@gmail.com (E.K.); 2 Department of Radiology, Shahida Islam Medical College, Bahawalpur 60600, Pakistan; naveeranaveed@gmail.com; 3 Department of Medicine, Sargodha Medical College, Sargodha 40100, Pakistan; zahra.riaz0913@gmail.com (Z.R.); roshaanfs19@gmail.com (R.F.); 4 Department of Medicine, Karachi Medical and Dental College, Karachi 74700, Pakistan; aemonaemon248@gmail.com (A.Q.); baigshehmeen@gmail.com (S.B.); 5 Department of Medicine, Allama Iqbal Medical College, Lahore 54550, Pakistan; drasawir44@gmail.com; 6 Department of Medicine, University College of Medicine and Dentistry (The University of Lahore), Lahore 54600, Pakistan; zoyamustunsar.1@gmail.com; 7 Department of Medicine, Dow University of Health Sciences, Karachi 74200, Pakistan; aayeeshhakkhaan@gmail.com; 8 Department of Medicine, Ziauddin Medical College, Karachi 75600, Pakistan; sadiajmangan@gmail.com; 9 Department of Medicine, Liaquat University of Medical and Health Sciences, Shahdadpur 76090, Pakistan; mehakkanyalal4@gmail.com; 10 Department of Internal Medicine, Multan Medical and Dental College, Multan 59300, Pakistan; 11 Department of Medicine, National heart and Lung Institute, Imperial College London, London SW7 2AZ, UK; ali.hasan21@imperial.ac.uk

**Keywords:** Neonatal Respiratory Distress Syndrome (NRDS), budesonide, pulmonary surfactant (PS)

## Abstract

*Background and Objectives:* Neonatal respiratory distress syndrome (NRDS), resulting from a deficiency of pulmonary surfactant (PS), can cause alveoli to collapse. Glucocorticoids reduce inflammation and are effective in reducing pulmonary swelling. This study aims to assess the effectiveness of the combination of PS and budesonide in the management of NRDS. *Materials and Methods:* Publications between 21 May and 24 November were screened through PubMed, Cochrane and Embase. Data analysis was performed on RevMan 5.3 software. Subgroup analysis was performed to evaluate the routes of administrations. *Results:* The use of budesonide along with pulmonary surfactant for treating NRDS revealed the following results: (1) a reduced duration of invasive mechanical ventilation (standardized mean difference (SMD) = −1.06, 95% confidence interval (CI) = −1.55 to −0.56, *p* < 0.0001); (2) reduced rate of bronchopulmonary dysplasia (BPD) occurrence (relative risk (RR) = 0.72, 95% CI = 0.60 to 0.86, *p* = 0.0003); (3) reduced duration for hospital admittance (SMD = −0.38, 95% CI = −0.64 to −0.11, *p* = 0.005). The occurrence of complications, i.e., sepsis, pneumothorax, retinopathy of prematurity (ROP), necrotizing enterocolitis (NEC), rate of mortality, hyperglycemia and intraventricular hemorrhage (IVH), was not significantly different among the intervention and comparison group except for patent ductus arteriosus (PDA) and pulmonary hemorrhage, with their incidence being higher in the control group (*p* = 0.002 and *p* = 0.05, respectively). *Conclusions:* The combination of pulmonary surfactant and budesonide decreases the occurrence of BPD, duration of mechanical ventilation, length of hospital stay and risk of pulmonary hemorrhage and PDA. It does not increase the risk of complications and death and is clinically safe.

## 1. Introduction

Neonatal respiratory distress syndrome (NRDS), also known as hyaline membrane disease, is a chief pulmonary deficit in premature infants due to their underdeveloped lungs and inadequate surfactant production [1]. Fetal lung development begins in the 3rd week of gestation and occurs in five stages, i.e., embryonic, pseudo glandular, canalicular, saccular and alveolar. The saccular stage occurs at 24–38 weeks and primarily includes the development of terminal sacs and type I and II pneumocytes necessary for the surfactant production that begins at 22 weeks of gestation. The preterm infants born before this span will have underdeveloped lung anatomy and insufficient surfactant production [2]. This results in alveolar collapse, difficult gas exchange and respiratory distress. The reason for this outcome is that, normally, the pulmonary surfactant forms a thin coating on the alveolar surface, where it decreases the surface tension, thus decreasing the work of breathing, preventing atelectasis and bronchiolar collapse. Also, it acts as a gateway for proper respiratory exchange and has an imperative role in the maintenance of innate immunity, and the surfactant also aids in the relaxation of the respiratory smooth muscles [3].

Several risk factors are registered that are responsible for elevating the rate of NRDS, including premature birth, which is the most remarkable, asphyxia, low birth weight, cesarean section delivery, multifetal pregnancy, diabetic mothers [4] and meconium aspiration syndrome [2].

Dyer J et al. [5] state that approximately 1% of all newborn babies worldwide suffer from NRDS, which gets significantly higher with a smaller gestational age. In the US, preterm babies with a gestational age between 26 and 30 weeks have a mortality rate of 50% to 93% due to NRDS. A study shows that NRDS is the principal reason for the neonatal intensive care unit (NICU) admissions of preterm infants [6] and is also the leading cause of morbidity and mortality in them [7].

BPD is a crucial and noteworthy complication of NRDS resulting from radical injury and inflammation triggered by mechanical ventilation and oxygen therapy in treatment, with a high morbidity and mortality rate along with neurological developmental defects during the initial years of life [8,9].

Corticosteroids, being anti-inflammatory, can be useful for intervention, but the practice of systemic ones is avoided due to their long-term deleterious effects [10]. Budesonide is an effective drug with evident curative outcomes, as it has a durable local anti-inflammatory action. Upon its intratracheal administration, it synthesizes intracellular fatty acid esters in the lining epithelium of the airway, which are hydrolyzed and retain the local anti-inflammatory action [9].

We conducted the research and a literature review, and so the results of this meta-analysis reveal that when budesonide is given along with surfactant, it shows an exceptionally better response in patients in comparison to surfactant alone. When budesonide is administered via the intratracheal route along with surfactant, it drastically enhances the respiratory action of infants, along with a substantial decrease in the rate of BPD and mortality compared to surfactant alone [11]. Surfactant not only aids in smooth and efficient drug delivery but also augments the improved solubility and absorption of budesonide [11,12]. Budesonide, when inhaled alone, reduced the incidence of BPD but at the expense of a high mortality rate, so in order to avoid such unpleasant events, it was given along with the surfactant, which reduced the incidence of BPD by 20% without any significant rise in mortality or any marked neurological deficit [10]. It was seen that this adjunct therapy, when administered via the endotracheal route, ameliorated the O_2_ saturation in NARDS and promoted healthier lung development [13].

Without appropriate management and treatment, a neonate with respiratory distress syndrome will have a poor gaseous exchange, resulting in hypoxemia and hypercapnia, which will ultimately lead to respiratory acidosis and metabolic acidosis with atypical blood gases. This will cause the baby to become indolent, flaccid and hypotensive [2].

To prevent NRDS in infants that are at risk, antenatal steroids are recommended, as they accelerate fetal lung development along type I and II pneumocytes and adequate surfactant production [14]. According to European Consensus Guidelines on the management of respiratory distress syndrome, the administration of antenatal steroids along with some other management therapies is recommended, as it improves the condition and decreases the mortality rate. But, any patient with spontaneous preterm labor should not be given steroids as they can cause some serious psychological and neurocognitive problems [15]. The main aim of this systematic review is to access the efficacy and benefits of pulmonary surfactant plus budesonide in terms of the duration of mechanical ventilation, length of hospital stay and BPD incidence due to NRDS in comparison to surfactant alone.

## 2. Methodology

This meta-analysis was conducted in accordance with the PRISMA 2020 (Preferred Reporting Items for Systematic Reviews and Meta-Analyses) guidelines No. (13) and the protocol were prospectively registered with PROSPERO (registration number: CRD420251077341). The study exclusively utilized previously published data, exempting it from requiring institutional review board approval.

### 2.1. Research Sources

Authentic databases including PubMed, Cochrane and Embase were searched from 21 May to 24 November. Two members scrupulously executed a screening of the studies for inclusion and exclusion purposes.

### 2.2. Search Method and Strategy

Our study pertains to the following keywords:

Bronchopulmonary dysplasia, neonatal respiratory distress syndrome, neonate respiratory distress syndrome, newborn respiratory distress syndrome, surfactant, pulmonary surfactants, budesonide, acute respiratory distress syndrome [Supplementary Table S1].

The above mentioned keywords were used on the database in the strategical form, i.e.,

(((Broncho pulmonary dysplasia) OR (Neonatal respiratory distress syndrome)) OR (Neonate respiratory distress syndrome)) OR (Acute respiratory distress syndrome)) AND ((Pulmonary surfactant) OR (Surfactant))) AND (Budesonide))).

### 2.3. Search Criteria

Specific criteria for the inclusion and exclusion of studies were devised. For inclusion, the criterion chiefly demands the following:

Primary outcomes:

- Randomized controlled trials that have shown a direct comparison of budesonide in combination with pulmonary surfactant as an intervention with pulmonary surfactant alone as a control group to treat bronchopulmonary dysplasia.
- Less than 28 weeks gestational age of preterm infants with a very low birth weight.
- Premature infants suffering from bronchopulmonary dysplasia.
- Studies revealing the duration of mechanical ventilation.
- The length of hospital stay.
- Bronchopulmonary dysplasia as a primary outcome.
- Secondary outcomes:
- Pneumothorax.
- Interventricular hemorrhage.
- Necrotizing enterocolitis.
- Patent duct arteriosus.
- Retinopathy of prematurity.
- Mortality rate.
- The exclusion criterion encompasses the following:
- Non-randomized controlled trials.
- Other corticosteroids combined with pulmonary surfactant.
- Studies without an abstract.
- Studies with a language barrier.

### 2.4. Study Selection

To identify relevant studies, we first screened the titles and abstracts of the collected data. Next, two researchers (U.F & E.K) independently reviewed the full texts of the shortlisted studies to determine their eligibility for inclusion in the meta-analysis. Any disagreements were resolved through discussions with a third researcher (M.K). To ensure transparency, the PRISMA guidelines (13) were used to outline the study selection process and document reasons for excluding certain studies.

### 2.5. Data Extraction

Data were carefully extracted from each selected randomized controlled trial (RCT), including baseline characteristics, intervention details and participant numbers. We also collected key outcomes in both continuous and categorical formats.

The primary outcomes include percentage of bronchopulmonary dysplasia (BPD) cases, duration of mechanical ventilation and length of hospital stay (in days).

The secondary outcomes include mortality rate, intraventricular hemorrhage (IVH), incidence of retinopathy of prematurity (ROP), cases of necrotizing enterocolitis (NEC), incidence of patent ductus arteriosus (PDA), hyperglycemia occurrence, sepsis, percentage of pneumothorax and cases of pulmonary hemorrhage.

### 2.6. Quality Assessment

The methodological quality of the included randomized controlled trials (RCTs) was assessed using the Cochrane risk of bias tool [16], which evaluates five domains: random sequence generation, allocation concealment, blinding of participants and personnel, blinding of outcome assessment and selective reporting. For random sequence generation, studies that reported adequate methods, such as computer-generated sequences or random number tables, were rated as low-risk, while those with inadequate or unclear methods were rated as high- or unclear-risk. Allocation concealment was judged based on whether adequate methods (e.g., opaque sealed envelopes or central randomization) were used to prevent selection bias. The blinding of participants and personnel was evaluated by determining whether procedures were in place to prevent knowledge of intervention assignments; studies without proper blinding were rated as higher-risk due to potential performance bias. The blinding of outcome assessment considered whether assessors were blinded to group assignments, particularly for subjective outcomes; a lack of blinding here led to high- or unclear-risk ratings. For selective reporting bias, we compared the reported outcomes in the final publication with those pre-specified in trial registries or protocols when available. Studies that omitted outcomes, selectively reported favorable results or lacked a publicly accessible protocol were considered as high- or unclear-risk. Each domain was rated as having a low, high or unclear risk of bias, and assessments were performed independently by two reviewers, with discrepancies resolved by consensus or consultation with a third reviewer.

### 2.7. Statistical Analysis

The data in this meta-analysis was analyzed using RevMan 5.2 (Review Manager v.5.2). All statistical methods applied followed a random-effects model using the inverse variance (IV) method for both dichotomous and continuous outcomes. Dichotomous data were expressed as relative risk (RR) or risk difference with a 95% confidence interval (CI), while continuous data were represented using mean difference (MD). The 95 percent CI provides the range within which we are 95 percent confident that the true RR lies; if this interval does not cross 1.0, the result is considered statistically significant at the *p* = 0.05 level. A relative risk closer to 1 or greater than 1 increases the risk in the intervention group. A *p*-value below 0.05 was considered statistically significant. Statistical heterogeneity was assessed visually through a forest plot using the χ^2^ and I^2^ tests. To identify the source of high heterogeneity, a leave-one-out analysis was performed. The wider the confidence interval, the greater the chances of heterogeneity. A relative risk closer to 1 or greater than 1 increases the risk in the intervention group.

## 3. Results

### 3.1. Study Selection

**Figure 1:**
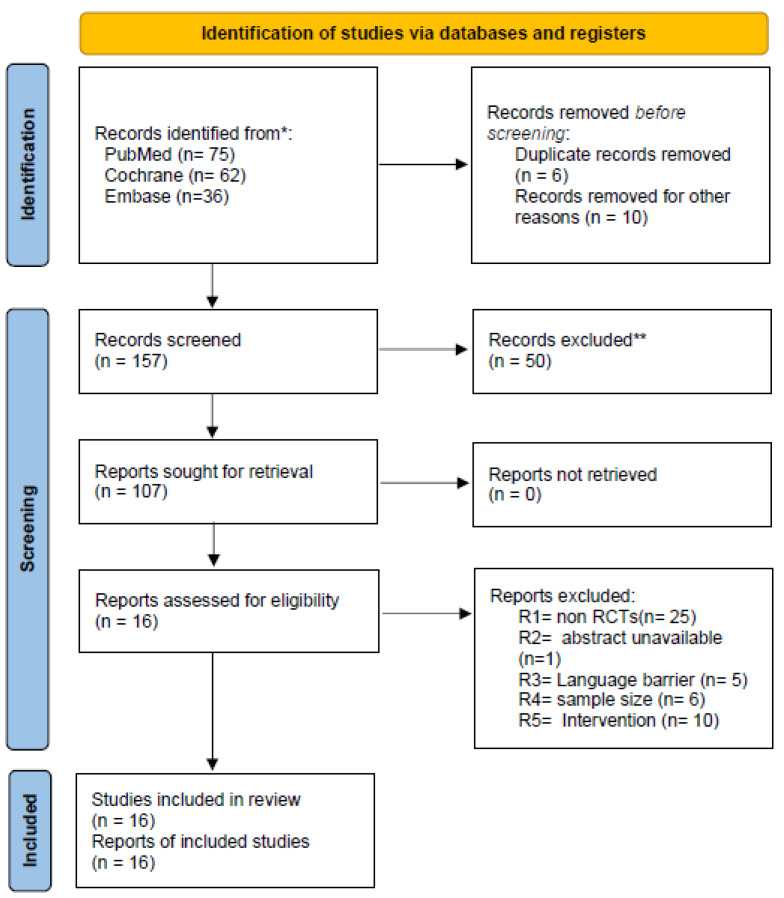
Flowchart for the search strategy and study selection process. * Database searches were performed using controlled vocabulary and free-text terms tailored to each database. ** Screening exclusions were based on study design, scope, or population misalignment with the eligibility criteria.

The search was made on authentic electronic databases that include PubMed, Cochrane and Embase. A total of 173 studies were retrieved by researchers meticulously. Out of those 173 studies, 6 duplicated studies were removed and 10 others were removed because they did not match our inclusion criterion. Two researchers scrupulously screened a total of 157 articles, out of which 50 were excluded due to study design, sample size, intervention used and language barrier. A total of 16 randomized controlled trials conformed to the inclusion criteria of this study [9,11,17,18,19,20,21,22,23,24,25,26,27,28,29,30]. A detailed selection process is presented in the PRISMA flowchart (Figure 1).

### 3.2. General Information

Intervention for bronchopulmonary dysplasia in preterm infants is the subject matter in this study. A total of 16 RCTs are included in our study, with the objective to use pulmonary surfactant combined with budesonide as an intervention [9,11,17,18,19,20,21,22,23,24,25,26,27,28,29,30]. Fourteen articles compared PS combined with budesonide with the PS-alone therapy [9,11,17,18,19,20,21,22,26,27,28,29,30]. These studies discussed a total of 2680 cases. The remaining two RCTs gave tracheal PS with atomization inhalational therapy of budesonide, with the control group treatment being pulmonary surfactant alone [24,25]. These two articles included a total of 200 cases. The baseline characteristics across studies show that enrolled preterm infants had gestational ages ranging from approximately 25 to 32 weeks and birth weights between 635 g and 1950 g, with no significant differences between trial and control groups as shown in Table 1.

### 3.3. Quality Assessment of Included Studies

In order to assess the merit of the conducted analysis, we used the Cochrane risk bias assessment tool. Every involved article followed the random sequence of grouping [17,18,19,20,21,22,23,24,25,26,27,28,29,30,31]. Eight papers reported the allocation concealment [9,17,18,20,21,26,28,30]. Four papers implemented the double-blind to prevent performance bias [9,26,30,31]. Only two studies executed a triple-blind to avoid detection bias [9,28]. A total of 11 out of 16 papers did not have any missing preset outcome indicators [17,18,19,20,21,22,23,24,25,26,27]. Five studies were at low risk of selective reporting [19,28,29,30] and nine were at high risk; however, one study was relatively unclear (Figure S1A,B)

### 3.4. Outcome of Combined Therapy on the Duration of Mechanical Ventilation for NRDS Treatment

**Figure 2:**
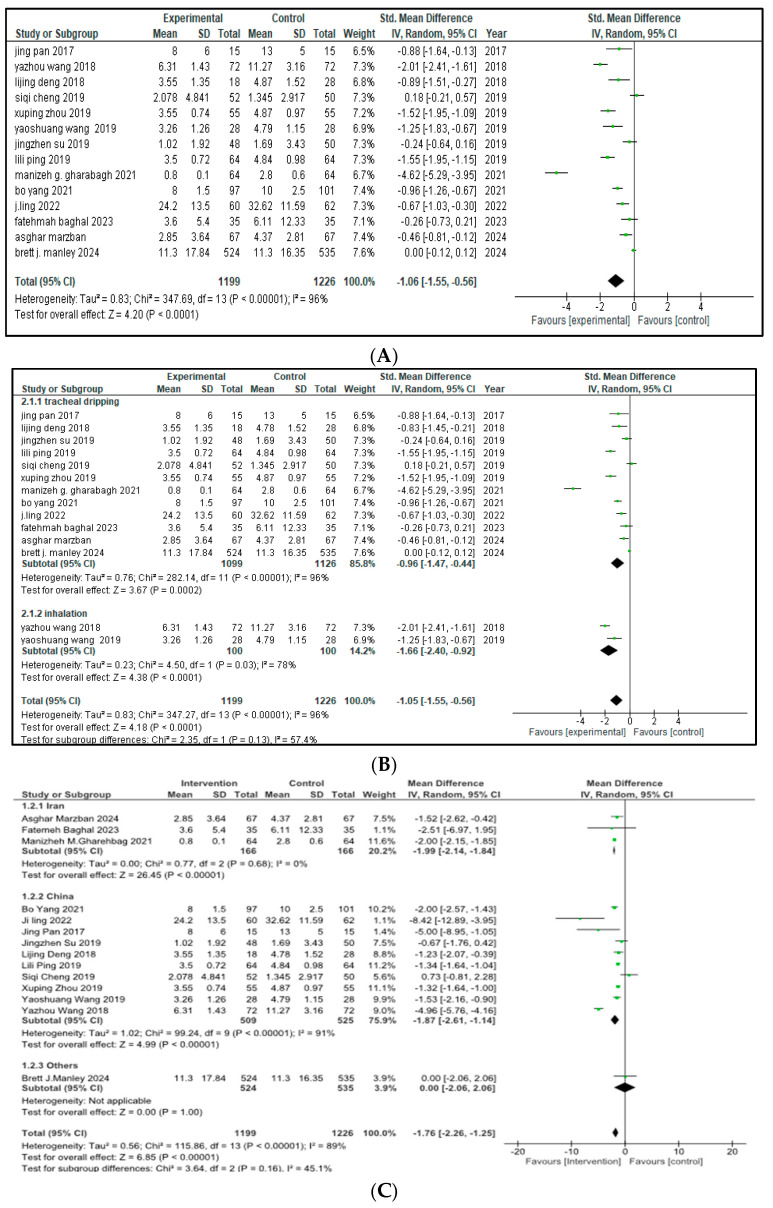
(**A**) Duration of mechanical ventilation [9,17,18,19,20,21,22,23,24,25,26,28,29,30] (**B**) Forest plot of subgroup analysis of invasive mechanical ventilation on the basis of route of administration (**C**) Forest plot of subgroup analysis of invasive mechanical ventilation on the basis of Geographical Area, China (I^2^ = 91%) contributed most heterogeneity than Iran (I^2^ = 0%).

Fourteen out of the sixteen included trials (total *n* = 2425 infant; 1199 in the intervention arm and 1226 in the control arm) stated the impact of intervention therapy on the duration of mechanical ventilation for NRDS treatment. Pooled analysis demonstrated that the combination of budesonide plus pulmonary surfactant significantly shortened the time on ventilator support compared to the control group (SMD = −1.06, 95% CI = −1.55 to −0.56, *p* < 0.0001), corresponding to an average of approximately 1.2 fewer days on ventilator support in the intervention group, as depicted in Figure 2.

The arithmetical outcomes revealed that *p* < 0.00001 and that there was a 95% confidence interval (CI) from −1.55 to −0.56. This interval does not cross zero, indicating a consistent benefit across studies. High heterogeneity was observed (I^2^ = 96%, χ^2^ = 347.69, *p* < 0.00001), and a leave-one-out sensitivity analysis did not identify any single study disproportionately influencing this result.

With respect to the distinct ways of drug administration, an analysis among subgroups was performed. Out of the fourteen studies, twelve used tracheal dripping (n = 2225; 1099 in the intervention group and 1126 in the control group) and two used inhalation as the route of drug administration (n = 200; 100 included in the intervention arm and 100 in the control arm). The results show that the tracheal dripping subgroup had a statistically significant difference in the duration of mechanical ventilation between the intervention and the control group (SMD = −0.96, 95% CI = −1.47 to −0.44, *p* = 0.0002). As for the subgroup in which the drug was administered through inhalation, a significantly shorter duration of mechanical ventilation was observed in the intervention group (SMD = −1.66, 95% CI = −2.40 to −0.92, *p* < 0.0001). The test for subgroup analysis did not reach statistical significance (*p* = 0.13), indicating no strong evidence of a differential effect by method of administration as presented in Figure 2B. Further stratification by geographical origin showed that studies originating from China contributed most to the heterogeneity (I^2^ = 91%) compared to studies from Iran (I^2^ = 0%), but the results remained insignificant as presented in Figure 2C.

### 3.5. Outcome of Combined Therapy on the Rate of BPD Occurrence for NRDS Treatment

**Figure 3:**
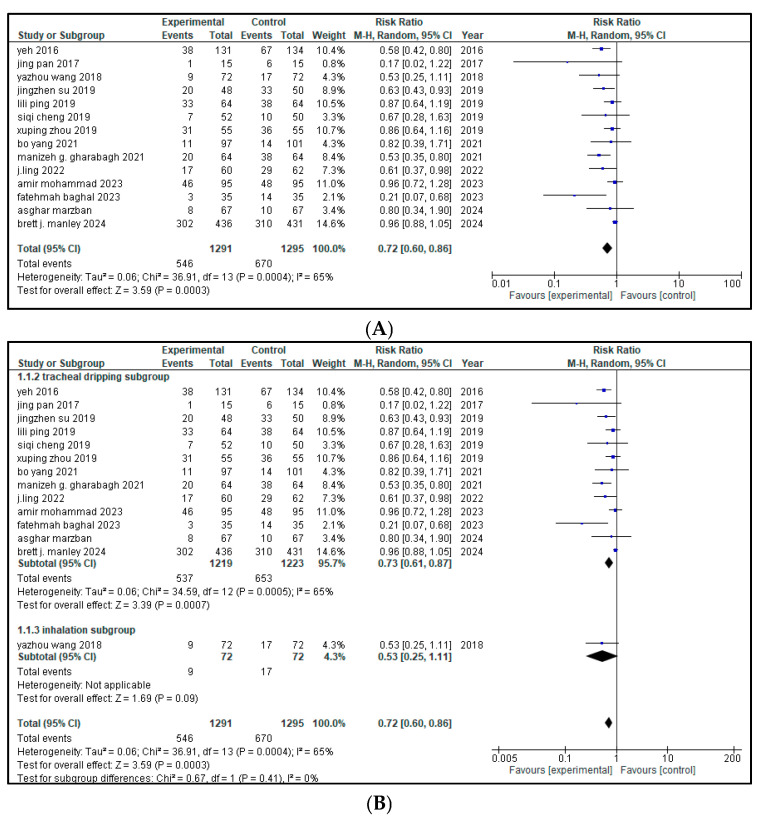
(**A**) forest plot demonstrating the pool risk ratio for the incidence of BPD [9,11,17,18,19,20,21,22,23,24,25,26,27,28,29,30] (**B**) Forest plot of subgroup analysis on the basis of route of administration.

Fourteen of the sixteen included articles (total n = 2586 infants; with 1291 in the intervention group and 1295 in the control group) stated the impact of intervention therapy on the rate of BPD cases among patients with neonatal respiratory distress syndrome (NRDS). A pooled analysis demonstrated a significant decrease in the rate of bronchopulmonary dysplasia (BPD) cases in the intervention group (RR = 0.72, 95% CI = 0.60 to 0.86, *p* = 0.0003) as shown in Figure 3. This 28% relative reduction translates into 28 fewer cases of BPD per 100 infants treated, suggesting a clinically meaningful benefit in reducing lung disease. The 95% CI does not include the null value of 1.0, reinforcing the reliability of this finding.

Moderate heterogeneity was present across studies (I^2^ = 65%), suggesting some degree of variability. A leave-one-out sensitivity analysis was conducted for high heterogeneity and it was significantly reduced by leaving out Brett J. Manley from I^2^ 65% to 33%. Based on the distinct ways of management, an analysis among the subgroups was performed. Out of the 14 included studies, 13 used tracheal dripping (n = 2442; 1219 in the intervention arm and 1223 in the control arm) and 1 used inhalation (n = 144; 72 in the intervention arm and 72 in the control arm) as the drug delivery route. The results show that the tracheal dripping subgroup had a statistically significant reduction in the rate of BPD cases in the intervention group (RR = 0.73, 95% CI = 0.61 to 0.87, *p* = 0.0007). As for the inhalation subgroup, a decreased rate of BPD cases was observed in the intervention group (RR = 0.53), though with a wide 95% CI (0.25 to 1.11) crossing the null and thus being non-significant. The test for subgroup difference showed that *p* = 0.41, suggesting no clear effect by route of administration, as presented in Figure 3B.

### 3.6. Outcome of Combined Therapy on the Duration of Hospital Admittance for NRDS Treatment

**Figure 4:**
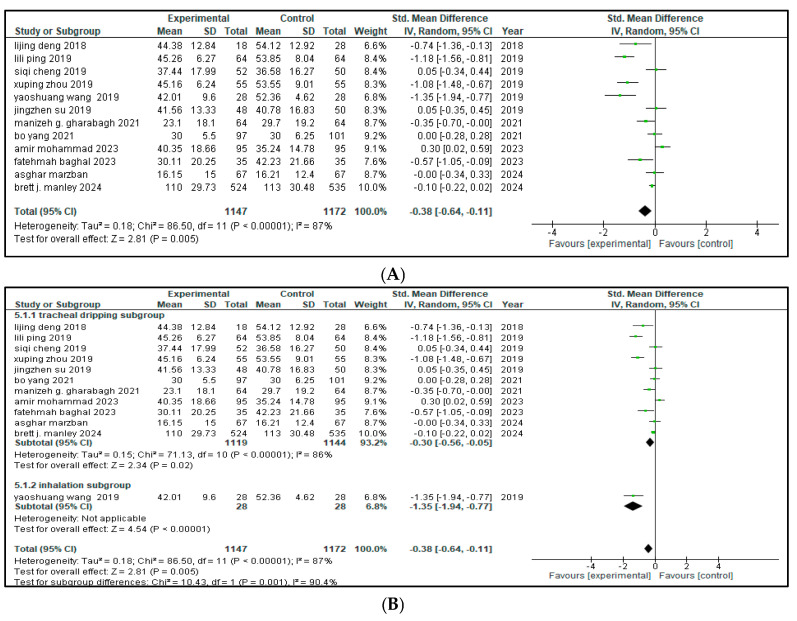

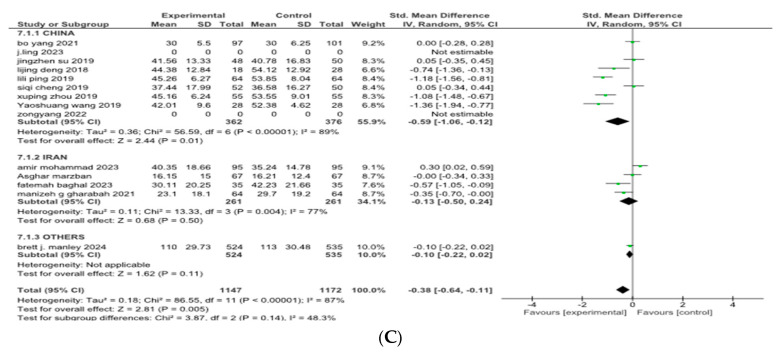
(**A**) Forest plot showing pooled analysis for length of hospital stay [9,17,18,20,21,22,23,25,26,27,28,30] (**B**) Forest plot of subgroup analysis on the basis of route of administration (**C**) Forest plot of subgroup analysis on the basis of geographical area, China has higher heterogeneity (I^2^ = 89%) than Iran (I^2^ = 77%).

Twelve of the sixteen included studies reported the effect of combined budesonide and pulmonary surfactant (PS) therapy on the duration of hospital admittance for neonatal respiratory distress syndrome (NRDS), involving a total of 2319 infants (1147 in the intervention group and 1172 in the control group). As shown in Figure 4, the pooled analysis indicated a statistically significant reduction in hospital stay among infants receiving the combination therapy (SMD = −0.38, 95% CI = −0.64 to −0.11; *p* = 0.005), suggesting a moderate beneficial effect in the combined therapy. The 95% CI lies entirely below zero, indicating a reliable treatment effect. The arithmetical results reveal that *p* < 0.00001, with substantial heterogeneity (I^2^ = 87%).

A leave-one-out sensitivity analysis was conducted to explore this high heterogeneity, but no single study significantly altered the results.

Then, a subgroup analysis was carried out based on different methods of drug administration. Out of the 12 included studies, 11 used tracheal dripping (n = 2263; 1119 in the intervention arm and 1144 in the control arm) and 1 used inhalation (n = 56; 28 in the intervention arm and 28 in the control arm) as the drug delivery route. The results show that the tracheal dripping subgroup had a statistically significant benefit (SMD = −0.30, 95% CI = −0.56 to −0.05, *p* = 0.02), where the 95% CI remained below zero. As for the inhalation subgroup, a significantly shorter duration of hospital admittance was observed in the intervention group (SMD = −1.35, 95% CI = −1.94 to −0.77, *p* < 0.00001), with a wide but still entirely negative 95% CI, proposing a beneficial treatment effect.

The test for subgroup difference was significant (*p* = 0.001), demonstrating a potential influence of route of administration on the duration of the hospital stay, as presented in Figure 4B. Further stratification by geographical origin showed that studies originating from China contributed most to the heterogeneity (I^2^ = 89%) compared to studies from Iran (I^2^ = 77%), but the results remained insignificant as presented in Figure 4C.

### 3.7. Secondary Outcome Indicators Among NRDS Patients

The related complications included in the study were sepsis, pneumothorax, retinopathy of prematurity (ROP), necrotizing enterocolitis (NEC), patent ductus arteriosus (PDA), rate of mortality, incidence of hyperglycemia, pulmonary hemorrhage and intraventricular hemorrhage (IVH). Most secondary outcomes did not exhibit statistically significant differences between the intervention group and the control group (as each of them has a *p* > 0.05), with low to moderate heterogeneity across studies.

However, patent ductus arteriosus (PDA) (RR = 0.83, *p* = 0.002) and pulmonary hemorrhage (RR = 0.70, *p* = 0.05) were significantly brought down in the experimental group, with narrow 95% confidence intervals and no heterogeneity (I^2^ = 0), conveying potentially meaningful clinical results.

Table 2 represents the summary of the aforementioned secondary outcomes. Figure 5, Figure 6, Figure 7, Figure 8, Figure 9, Figure 10, Figure 11, Figure 12 and Figure 13 refer to the analytical details of all secondary outcomes.

## 4. Discussion

### 4.1. Summary of Findings

Our meta-analysis, gathering data from different RCTs, offers a rigorous comparison of PS combined with budesonide versus PS alone in the management of NRDS and the prevention of BPD in preterm infants. It corroborates previous research and also identifies notable differences. The findings designate that the combined therapy leads to a statistically significant reduction in the incidence of BPD, mechanical ventilation duration and hospital stay. Notably, both tracheal instillation and inhalation methods demonstrated efficacy, with inhalation showing a more conspicuous impact on reducing the duration of hospitalization and ventilation, while tracheal instillation was more effective in reducing BPD rates. Moreover, the intervention was associated with a reduced risk of pulmonary hemorrhage. However, there was no significant variation in mortality, hyperglycemia or IVH between the two groups. Secondary complications, including sepsis, pneumothorax, ROP and NEC, showed no statistically significant difference, except PDA, where a notable difference was detected. These results bring out the potential benefits of PS and budesonide combined therapy in improving respiratory outcomes in preterm infants. At the same time, it emphasizes the need for further investigations regarding its long-term safety and effectiveness.

### 4.2. Comparison with Prior Literature

**Figure 10:**
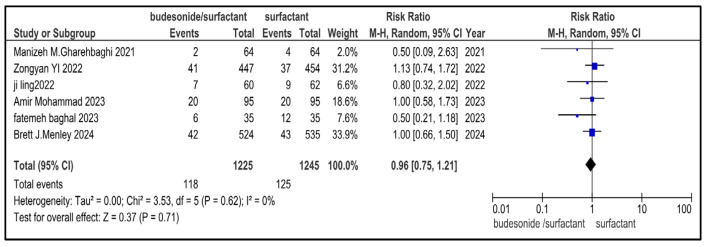
Forest plot showing pooled analysis of the incidence of NEC in infants with NRDS, comparing the intervention and the control group, with RR = 0.96 (95% CI: 0.75 to 1.21 *p* = 0.71), indicating a statistically non-significant lower risk in the intervention group [9,26,27,29,30]. Moreover, no heterogeneity (I^2^ = 0%) was observed across included studies. A random-effects model was used for calculating the pooled results. Note: blue squares represent individual study estimates and black diamonds represent the pooled summary effect, while the horizontal lines represent 95% confidence intervals. NRDS, neonatal respiratory distress syndrome; NEC, necrotizing enterocolitis; CI, confidence interval.

**Figure 11:**
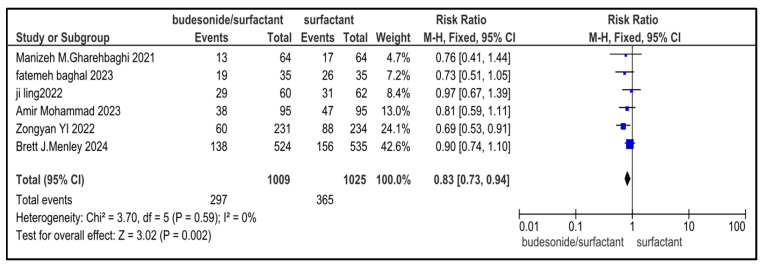
Forest plot demonstrating pooled analysis of the incidence of PDA in infants with NRDS, comparing the intervention and the control group, with RR = 0.83 (95% CI: 0.73 to 0.94, *p* = 0.002), indicating a statistically significant lower risk in the intervention group [9,26,27,29,30]. Moreover, no heterogeneity (I^2^ = 0%) was observed across included studies. A random-effects model was used for calculating the pooled results. Note: blue squares represent individual study estimates and black diamonds represent the pooled summary effect, while the horizontal lines represent 95% confidence intervals. NRDS, neonatal respiratory distress syndrome; PDA, patent ductus arteriosus; CI, confidence interval.

**Figure 12:**
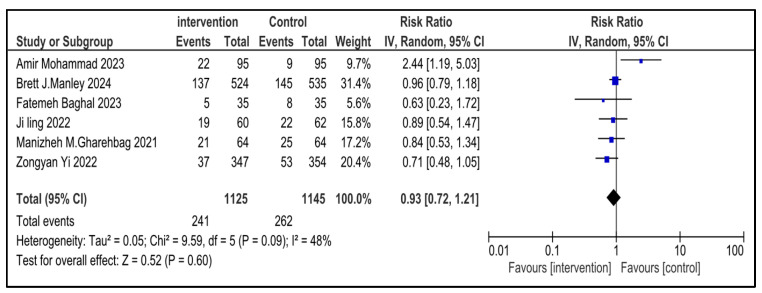
Forest plot demonstrating pooled analysis of the incidence of sepsis in children with NRDS, comparing the intervention and the control group, with RR = 0.93 (95% CI: 0.72 to 1.21, *p* = 0.60), indicating a statistically non-significant lower risk in the intervention group [9,26,27,29,30]. Moreover, moderate heterogeneity (I^2^ = 48%) was observed across included studies. A random-effects model was used for calculating the pooled results. Note: blue squares represent individual study estimates and black diamonds represent the pooled summary effect, while the horizontal lines represent 95% confidence intervals. NRDS, neonatal respiratory distress syndrome; IV, inverse variance; CI, confidence interval.

In our meta-analysis, the intervention group substantiated a reduced duration of mechanical ventilation and hospitalization, along with a lower incidence of BPD. This is accordant with the study of Zongyan Yi et al., indicating that the combination of PS and budesonide can enhance the pulmonary function of children with NRDS so that the time for mechanical ventilation, length of hospital stay and BPD can be minimized [31]. Meng SS et al. reported that pulmonary surfactant keeps alveolar tension low and balances the alveolar volume, increases gas exchange and reduces edema in alveoli, as well as cutting back local mechanical forces and systemic inflammatory reactions in acute respiratory distress syndrome (ARDS) [32]. This coincides with our study, indicating that the intervention is associated with a reduced mortality rate and BPD incidence. In addition, Barrette AM et al. highlighted that BPD, a chronic lung disease with long-term complications, arises in premature infants partly from inflammatory changes in the lungs. Delivering budesonide suspended in surfactant effectively attenuates the incidence of BPD [33]. This is consistent with our meta-analysis, indicating that the combination of budesonide and PS has a positive impact on BPD incidence. The statistical analysis of mortality rates, complications and risk of hemorrhage in the intervention and control group shows no significant differences, as shown in Figure 10, Figure 11 and Figure 12. As the risk of mortality and complications is not increased, this combination is relatively safe. Hence, it can be used by clinicians in the management of NRDS. A key strength of this meta-analysis is the significant reduction in the incidence of pulmonary hemorrhage in the intervention group compared to the control group, accentuating the potential benefits of this therapy.

Our meta-analysis assessed 16 studies, all of which used budesonide as an inhaled preparation. However, the routes of administration varied: 2 studies used aerosol inhalation as mode of administration, while the remaining 14 studies used an intratracheal method. According to the analysis of different administration routes, the subgroup analysis showed that the duration of invasive mechanical ventilation in the inhalation subgroup was shorter in the intervention group than in the control group, as demonstrated in Figure 2A. Similarly, in the inhalation subgroup, the length of hospital stay was significantly shorter in the intervention group than in the control group, as shown in Figure 4A. Taking into account the occurrence of BPD, the subgroup with the tracheal instillation of budesonide showed a significant reduction in its occurrence. Based on these findings, it is evident that severe NRDS can be effectively handled with the combination of budesonide and surfactant therapy. This strategy not only alleviates lung conditions in neonates but also improves the histological structure of lungs. A Gharehbaghi MM et al. demonstrated that the intratracheal administration of a combination of surfactant and budesonide enhances short-term outcomes in preterm infants. This approach reduces the occurrence of bronchopulmonary dysplasia (BPD), decreasing the need for multiple doses of surfactant replacement therapy. BPD was diagnosed in 48.4% neonates, with 59.4% in the surfactant group and 31.3% in the surfactant + budesonide group, which supports our study [34]. The early intratracheal administration of budesonide, using surfactant as a carrier, led to a lower risk of mortality or chronic lung disease. Wang J et al., in an observational study, found that the intratracheal instillation of budesonide can greatly improve lung function, shorten the incidence of BPD and minimize the need for the repeated use of PS, which further aligns with our study. The intratracheal instillation of budesonide and pulmonary surfactant provides a high initial bioavailability in the lungs. Chen CM et al. studied that the use of inhaled glucocorticoids in preterm infants is mechanically demanding and operationally intricate, and that it also yields constrained outcomes.

On the other hand, the intratracheal administration of PS and budesonide suspension has been shown to significantly reduce the incidence of BPD or mortality while posing no immediate adverse effects in very-low-birth-weight infants with severe RDS [7]. However, the use of intratracheal budesonide combined with pulmonary surfactant in extremely preterm infants did not lead to a higher risk of fatality or detrimental physical or neurological outcomes [11,35,36]. In a meta-analysis, Tang W et al. indicated that the early airway delivery of a combination of budesonide and PS could shorten the duration of assisted ventilation, invasive ventilation and hospital stays in preterm infants with RDS. But, its subgroup analysis based on the method of budesonide administration (inhalation versus intratracheal instillation) revealed that the intratracheal instillation subgroup showed reductions in mortality, assisted ventilation time and hospital stays compared to the inhalation subgroup [37]. At the same time, Bassler D et al. found that inhaled budesonide may increase the mortality rate in preterm infants, with 82 deaths (19.9%) among 413 infants compared to 58 deaths (14.5%) among 400 infants [38]. The comparison between the tracheal instillation and inhalation of budesonide for treating NRDS signifies differences in curative outcomes. The studies suggest that tracheal instillation, when combined with surfactant, provides significant advantages, such as alleviating bronchopulmonary dysplasia (BPD) occurrence and improving the lung histological structure in preterm infants. Inhalation methods, though less invasive, may offer limited curative effects in severe cases of NRDS.

### 4.3. Risks and Safety Profile

While our findings underscore clinical benefits of budesonide when used in combination with pulmonary surfactant, it is necessary to assess and acknowledge the potential risks. Budesonide, as a potent corticosteroid, has known risks, including adrenal suppression, increased susceptibility to infections, impaired growth and neurodevelopmental impacts, especially if systemic absorption occurs. Although none of the included studies reported any adverse neurodevelopmental outcomes, it is important to note that many of these studies had limited sample sizes and duration, which may be insufficient for detecting late emerging effects. Therefore, longer follow-up studies are warranted for the evaluation of long-term safety profiles. Additionally, sparse data exist on the pharmacokinetic characteristics of budesonide in neonates, and its systemic bioavailability after intratracheal administration remains incompletely understood. More reliable data are required to clarify the extent to which budesonide enters systemic circulation and its potential implications. Until such data are available, clinicians should remain wary and weigh both the potential therapeutic benefits and the risks when considering this intervention.

### 4.4. Limitations

This meta-analysis has some limitations that should be acknowledged. First, a considerable portion of the studies were conducted in China, which limits the generalizability of the outcomes to other healthcare settings that have different clinical practices, standards of care and population characteristics. Second, there were remarkable variations in dosing regimens, techniques of delivery for budesonide and pulmonary surfactant and the timing of administration, which may introduce clinical and methodological heterogeneity. Third, not all included studies were blinded, which increases the risk of performance or detection bias, leading to an overestimation of treatment effects. Fourth, many studies lacked long-term follow-ups, which limits the efficacy and safety of intervention, especially regarding neurodevelopmental and growth outcomes. Finally, publication bias remains a significant concern because studies that report negative or non-significant findings are less likely to be indexed, potentially skewing outcomes.

### 4.5. Future Research Directions

Future studies should focus on multicenter, randomized, placebo-controlled trials using standardized dosing regimens and stratification by gestational age, severity of NRDS and birth weight. Long-term monitoring is essential for assessing not only respiratory benefits but also neurodevelopment impacts, physical growth and adrenal function. Active safety monitoring systems should be established to identify late-onset complications such as immune suppression, metabolic disturbance and impaired hypothalamic–pituitary–adrenal (HPA) axis function. Additionally, comparative trials are required to evaluate the efficacy and safety of intratracheal vs. inhaled delivery methods, supported by pharmacokinetic and pharmacodynamic profiles. Studies should also explore cost-effectiveness, ease of administration and the role of non-invasive delivery methods in low-resource settings. Incorporating real-world data, including data from neonatal databases, may improve the external relevance of findings and support evidence-based guidelines.

## 5. Conclusions

Based on the results of this systematic review and meta-analysis, we conclude that the pulmonary surfactant combined with budesonide decreases the incidence of BPD, duration of mechanical ventilation, length of hospital stay and risk of pulmonary hemorrhage and PDA. Also, it does not increase the risk of complications and death; thus, it can be used clinically as a safe combination.

## Figures and Tables

**Figure 5:**
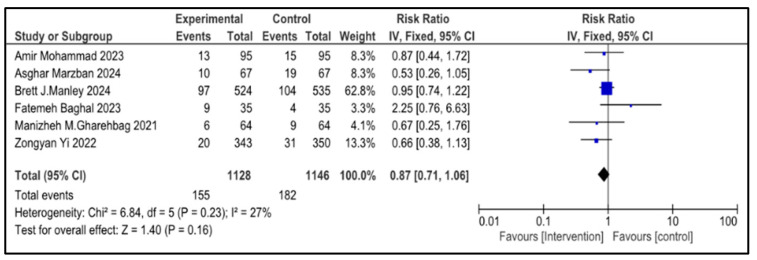
Forest plot showing pooled risk ratio (RR = 0.87), indicating a 13% reduction in the rate of mortality of infants with NRDS in the intervention compared to the control group [9,26,27,28,29,30]. The statistical analysis used a random-effects model and results were statistically non-significant (*p* = 0.23, 95% CI = 0.71 to 1.06) with a low heterogeneity (I^2^ = 27%). Note: blue squares represent individual study estimates and black diamonds represent the pooled summary effect, while the horizontal lines represent 95% confidence intervals. NRDS, neonatal respiratory distress syndrome; IV, inverse variance; CI, confidence interval.

**Figure 6:**
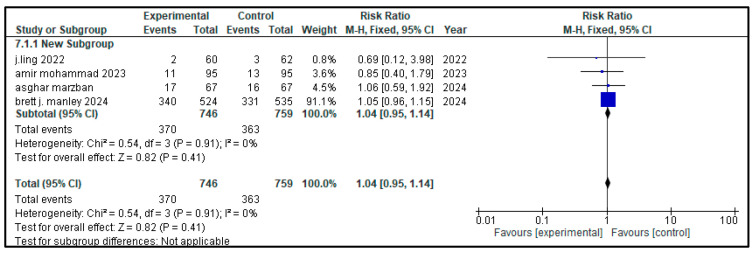
Forest plot showing pooled risk ratio (RR = 1.04) for the incidence of hyperglycemia in infants with NRDS, comparing the intervention group to the control group [9,27,28,29]. The analysis used a random-effects model and showed that the results were statistically non-significant (*p* = 0.91, 95% CI = 0.95 to 1.14) with a low heterogeneity (I^2^ = 0%) Note: blue squares represent individual study estimates and black diamonds represent the pooled summary effect, while the horizontal lines represent 95% confidence intervals. NRDS, neonatal respiratory distress syndrome; CI, confidence interval.

**Figure 7:**
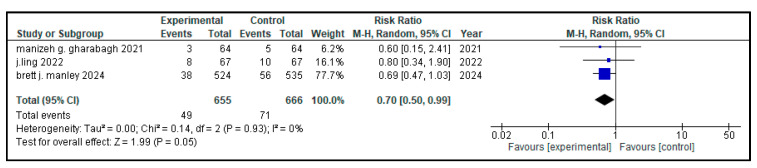
Forest plot showing the pooled analysis of the incidence of pulmonary hemorrhage in children with NRDS, comparing the intervention group to the control group [9,29,30]. A random-effects model was used and the results showed that there was a statistically significant lower risk of pulmonary hemorrhage in the intervention group (RR = 0.70, 95% CI = 0.50 to 0.99, *p* = 0.05). No heterogeneity was observed across included studies (I^2^ = 0%). Note: blue squares represent individual study estimates and black diamonds represent the pooled summary effect, while the horizontal lines represent 95% confidence intervals. NRDS, neonatal respiratory distress syndrome; CI, confidence interval.

**Figure 8:**
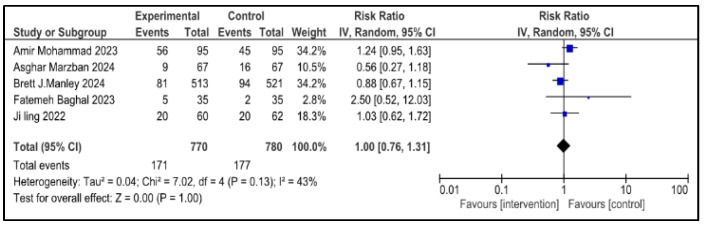
Forest plot showing pooled analysis of the incidence of IVH in infants with NRDS, comparing the intervention group to the control group, with RR = 1.00 (95% CI: 0.76 to 1.31 *p* = 1.00), indicating no measurable difference in risk between the intervention and control groups [9,26,27,28,29]. A random-effects model was used and the results were statistically non-significant, with moderate heterogeneity observed (I^2^ = 43%). Note: blue squares represent individual study estimates and black diamonds represent the pooled summary effect, while the horizontal lines represent 95% confidence intervals. NRDS, neonatal respiratory distress syndrome; IVH, intraventricular hemorrhage; IV, inverse variance; CI, confidence interval.

**Figure 9:**
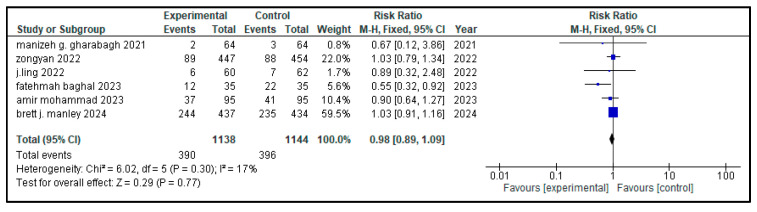
Forest plot depicting pooled analysis of incidence of ROP in children with NRDS, comparing the intervention and the control group, with RR = 0.98 (95% CI: 0.89 to 1.09 *p* = 0.77), indicating a statistically non-significant lower risk in the intervention group [9,26,27,29,30]. Moreover, low heterogeneity (I^2^ = 17%) was observed across included studies. The pooled results were calculated using a random-effects model. Note: blue squares represent individual study estimates and black diamonds represent the pooled summary effect, while the horizontal lines represent 95% confidence intervals. NRDS, neonatal respiratory distress syndrome; ROP, retinopathy of prematurity; CI, confidence interval.

**Figure 13:**
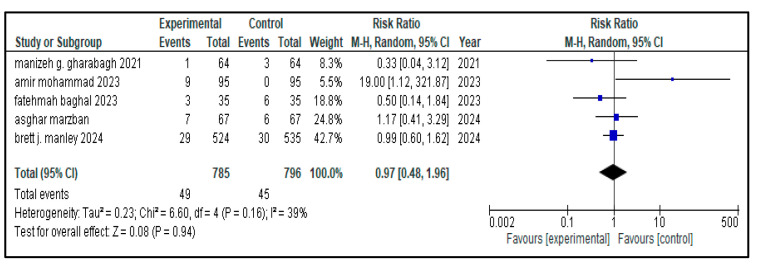
Forest plot showing pooled analysis of the incidence of pneumothorax in children with NRDS, comparing the intervention group and the control group, with RR = 0.97 (95% CI: 0.48 to 0.96, *p* = 0.94), indicating a statistically non-significant lower risk in the intervention group [9,26,27,28,30]. Moreover, moderate heterogeneity (I^2^ = 39%) was observed across included studies. A random-effects model was used for calculating the pooled results. Note: blue squares represent individual study estimates and black diamonds represent the pooled summary effect, while the horizontal lines represent 95% confidence intervals. NRDS, neonatal respiratory distress syndrome; CI, confidence interval.

**Table 1:** Baseline characteristics.

Author and Publication Year	T/C(n)	Gestational Weeks		Weight of Birth (g)		Intervention	
		Trial Group	Control	Trial Group	Control	Usage s Dosage (Trial)	Usage s Dosage (Control)
Yeh [11], 2016	131/134	26.5 ± 2.2	26.8 ± 2.2	882 ± 246	635 ± 283	PS (poractant alfa injection 100 mg/kg) with budesonide (0.25 mg/kg), tracheally	PS (poractant alfa injection 100 mg/kg) tracheally
Siqi Chen [17], 2016	52/50	30.84 ± 1.78	31.03 ± 1.66	1556.54 ± 350.44	1586.40 ± 462.71	PS (poractant alfa injection 100 mg/kg, 150–200 mg/kg) with budesonide (0.25 mg/kg), tracheally	PS (poractant alfa injection 150–200 mg/kg) tracheally
Bo Yang [18], 2021	67/101	30.6 ± 1.6	30.8 ± 1.7	1535 ± 351	1540 ± 340	PS (phospholipid of bovine 88.2 ± 37.3 mg/kg) with budesonide (0.25 mg/kg), tracheally	PS (phospholipid of bovine 88.5 ± 44.6 mg/kg) tracheally
Jing Pan [19], 2017	15/15	26.5 ± 1.8	30.0 ± 1.7	1260 ± 240	1360 ± 370	PS (phospholipid of bovine 70 mg/kg) with budesonide (0.25 mg/kg), tracheally	PS (phospholipid of bovine 70 mg/kg) tracheally
Lijing Deng [20], 2018	18/28	<37	<37	<1500	<1500	PS (poractant alfa injection 150 mg/kg) with budesonide (0.25 mg/kg), tracheally	PS (poractant alfa injection 150 mg/kg) tracheally
Jingzhen Su [21], 2016	48/50	26.68 ± 1.55	26.16 ± 1.45	1351.35 ± 337.77	1211.8 ± 267.78	PS (poractant alfa injection 200 mg/kg) with budesonide (0.25 mg/kg), tracheally	PS (poractant alfa injection 200 mg/kg) tracheally
Yiping Zhou [22], 2016	55/55	26.37 ± 1.22	26.43 ± 1.25	1287.14 ± 206.25	1256.84 ± 204.81	PS (poractant alfa injection 150 mg/kg) with budesonide (0.25 mg/kg), tracheally	PS (poractant alfa injection 150 mg/kg) tracheally
Lili Ping [23], 2016	64/64	26.1 ± 1.24	28.63 ± 1.2	1264.63 ± 207.12	1260.33 ± 205.87	PS (poractant alfa injection 150 mg/kg) with budesonide (0.25 mg/kg), tracheally	PS (poractant alfa injection 150 mg/kg) tracheally
Yazhou Wang [24], 2018	72/72	31.42 ± 4.27	31.51 ± 4.16	1634.54 ± 282.26	1672.54 ± 275.34	PS (poractant alfa injection 100 mg/kg) tracheally with budesonide (0.25 mg/kg), aerosol inhalation, 3 days	PS (poractant alfa injection 100 mg/kg) tracheally
Yaoshuang Wang [25], 2016	28/28	26.51 ± 0.23	26.46 ± 0.27	1320 ± 150	1260 ± 210	PS (phospholipid of bovine 70 mg/kg) tracheally, with budesonide (0.5 mg + 2 mL normal saline aerosol inhalation, 3 days	PS (phospholipid of bovine 70 mg/kg) tracheally
Fatemeh Baghal [26], 2023	35/35	29.94 ± 2.11	29.34 ± 2.19	1186 ± 224.14	1139.86 ± 230.28	PS 2.5 cc/kg of curosurf solution (3 cc/240 mg curosurf) with budesonide 250 µ/kg of palmicort from a vial of 0.25 mg/ml	PS 2.5 cc/kg of curosurf solution (3 cc/240 mg curosurf)
Amir Mohammad [27], 2023	95/95	28.94 ± 1.57	29.01 ± 1.57	1134.97 ± 237.61	1190 ± 289.33	PS 200 mg/kg for the initial dose and 100 mg/kg for subsequent doses with budesonide instilled once at a dose of 0.25 mg/kg	PS 200 mg/kg for the initial dose and 100 mg/kg for subsequent doses
Brett J. Manley [9], 2024	524/535	25.7	25.6	768 (634–900)	740 (624–910)	PS (poractant alfa) 200 mg/kg for first dose(100 mg/kg for second dose, if used) with 1 or 2 doses of budesonide 0.25 mg/kg	PS poractant alfa) 200 mg/kg for first dose (100 mg/kg for second dose, if used)
Asghar Marzban [28], 2024	67/67	31.66 ± 2.84	31 ± 2.96	1584.55 ± 505.02	1465.45 ± 520.88	PS intratracheal curosurf at a dose of 2.5 cc/kg with intratracheal administration of budesonide at a dose of 0.25 mg or 1 cc/kg	PS intratracheal curosurf surfactant alone at a dose of 2.5 cc/kg
Ji ling [29], 2022	60/62	29.6 ± 1.3	29.3 ± 1.4	1199 ± 137	1175 ± 175	PS first dose 200 mg/kg with budesonide 0.25 mg/kg mixed and intratracheal instillation added each time	PS alone, first dose 200 mg/kg
Manizheh M. Gharehbaghi [30], 2021	64/64	28.2 ± 1.7	28.4 ± 1.5	1055 ± 192	1089 ± 168	Intratracheal instillation of a mixed suspension of budesonide (pulmicort nebulizing suspension, AstraZeneca AB, Sodertalje, Sweden) 0.25 mg/kg and curosurf 200 mg/kg/ dose mixed in a single syringe	Intratracheal curosurf (Poractant alpha, Chiesi Farmaceutici, Italy) 200 mg/kg/dose (2.5 mL/kg/dose) after premedication with fentanyl 1–2 mic/kg

**Table 2:** Secondary outcomes.

Outcome Indicators	Articles	Heterogeneity	Experimental Group	Control Group	RR (95% CI)	*p*
Mortality	6	*p* = 0.23 I^2^ = 27%	155/1128	182/1146	0.87 (0.71, 1.06)	0.16
Hyperglycemia	4	*p* = 0.91 I^2^ = 0%	370/746	363/759	1.04 (0.95, 1.14)	0.41
Pulmonary Hemorrhage	3	*p* = 0.93 I^2^ = 0%	49/655	71/666	0.70 (0.50, 0.99)	0.05
IVH	5	*p* = 0.13 I^2^ = 43%	171/770	177/780	1.00 (0.76, 1.31)	1.00
ROP	7	*p* = 0.30, I^2^ = 17%	390/1138	396/1144	0.98 (0.89, 1.09)	0.77
NEC	6	*p* = 0.62, I^2^ = 0%	118/1225	125/1245	0.96 (0.75, 1.21)	0.71
PDA	6	*p* = 0.59, I^2^ = 0%	297/1009	365/1025	0.83 (0.73, 0.94)	0.002
Sepsis	6	*p* = 0.09, I^2^ = 48%	241/1125	262/1145	0.93 (0.72, 1.21)	0.60
Pneumothorax	5	*p* = 0.16, I^2^ = 39%	49/785	45/796	0.97 (0.48, 1.96)	0.94

Abbreviations: RR = risk ratio; CI = confidence interval; IVH: intraventricular hemorrhage (risk at 3–4 days); ROP: retinopathy of prematurity (risk at 4–5 weeks); NEC: necrotizing enterocolitis (risk at 2 to 4 weeks); PDA: patent ductus arteriosus (risk at 4–5 days).

## Data Availability

All data generated or analyzed during this study are included in this published article [and its Supplementary Information files].

## Supplementary Materials

The following supporting information can be downloaded at: https://www.mdpi.com/article/10.3390/medicina61081329/s1. Reference [39] is cited in the supplementary materials.
